# The combined effect of pulsed electric field treatment and brine salting on changes in the oxidative stability of lipids and proteins and color characteristics of sea bass (*Dicentrarchus labrax*)

**DOI:** 10.1016/j.heliyon.2021.e05947

**Published:** 2021-01-12

**Authors:** Janna Cropotova, Silvia Tappi, Jessica Genovese, Pietro Rocculi, Marco Dalla Rosa, Turid Rustad

**Affiliations:** a Department of Biotechnology and Food Science, Norwegian University of Science and Technology, Trondheim, Norway; b CIRI - Interdepartmental Centre of Industrial Agri-food Research, Alma Mater Studiorum University of Bologna, Campus of Food Science, Cesena, Italy; c Department of Agricultural and Food Sciences, Alma Mater Studiorum University of Bologna, Campus of Food Science, Cesena, Italy

**Keywords:** Sea bass, Brine salting, Oxidation reactions, Pulsed electric field, Color characteristics

## Abstract

A combined effect of pulsed electric field application and salting in a brine with 5 and 10% w/w NaCl on oxidative stability of lipids and proteins, as well as color characteristics of sea bass samples, was assessed in the study. The applied intensity of the current was set at 10 and 20 A corresponding to 300 and 600 V cm^−1^, respectively. Pulsed electric field (PEF) treatment led to a significant (p < 0.05) increase in primary and secondary lipid oxidation products expressed as peroxide value, conjugated dienes and 2-thiobarbituric acid reactive substances in PEF-treated samples compared to untreated ones. Conjugated dienes, as unstable primary oxidation products, correlated with b∗-value (p < 0.05, R = 0.789), suggesting their contribution to the yellowness of the fish flesh due to fast decomposition and conversion into secondary oxidation products yielding yellow pigmentation.

However, none of the fish samples treated at the higher current intensity of 20 A exceeded the acceptable level of 5 meq active oxygen/kg lipid according to the requirements of the Standard for fish oils CODEX STAN 329–2017, suggesting acceptable oxidative status quality of sea bass samples after the treatment. PEF-treated fish samples also showed a significant increase in Schiff bases and total carbonyls on day 5 and day 8 of brine salting compared to non-treated samples, revealing a strong effect of electroporation on protein oxidation.

## 1 Introduction

Salting is one of the oldest techniques used for fish preservation. Nevertheless, despite the emergence of other effective preservation methods, where freezing is the most common, salting of fish still remains popular among food producers and consumers. Scandinavian countries export large volumes of salted fish products to Italy, Spain, Portugal and Latin America (Thorarinsdottir et al., 2004). However, a wide variety of salted fish products including anchovies, sea bass, sardines, etc are currently produced and marketed in the Mediterranean Basin countries as well. Due to high palatability and acceptable price in the market, these product commodities have become popular and highly appreciated in many European and non-European countries. At the same time, due to global trends directed towards a healthy lifestyle and policies to overall reduction of salt in foods (WHO, 2016), consumers are increasingly demanding lightly salted fish products with good sensory properties (Fan et al., 2014). Therefore, lightly salted fish products have recently started to gain increased popularity in Europe.

Brine salting can be used individually for preparation of lightly salted products, or as a preliminary step in the production of heavily salted or cured-salted fish. The main ingredient used in brine salting is sodium chloride (NaCl). Even at relatively low concentrations (<5% w/w), it acts as a preservative inhibiting bacterial growth and deactivating enzymes by dehydration and osmotic pressure (Lupín et al., 1981). Other ingredients such as spices, herbs, sugar or antioxidants can also be used in the brine salting to improve sensory characteristics of the final product after salting. During brine salting, fish is submerged in a brine with concentration of salt 5–15% NaCl for 1–8 days. After this step, the fish fillets can be removed from the brine and placed with alternate thin layers of salt into stacks for 10–12 days to perform dry salting (Thorarinsdottir et al., 2004).

Many factors may affect the quality of the end product, including the type and quality of the fish raw material, concentration of salt in the brine and duration of brine salting (Thorarinsdottir et al., 2004). The salting method also influences the salt uptake by the muscle and affects its structural and mechanical properties. Thus, the rate of salt penetration into the fish muscle is higher during brine salting compared to dry salting (Akse et al., 1993).

Different pre-treatments have previously been studied to accelerate salt uptake by the muscle, including high intensity ultrasound brining (Chemat et al., 2011), pulsed vacuum brining (Andres et al., 2002), and vacuum tumbling (Mathias et al., 2003). At the same time, to the best of our knowledge, no studies have so far been performed on pulsed electric field (PEF) applications for brine salting of pelagic fish. PEF, as an emerging non-thermal technology, has high potential to contribute to enhanced diffusion of salt into the fish muscle through cell permeabilization leading to increased mass transfer (Hafsteinsson et al., 2000). PEF is based on the application of short duration pulses (few μs to ms) of an electric field (0.1–50 kV/cm) to a sample placed between two electrodes. Through a phenomenon known as electroporation, PEF allows to modify, in a reversible or irreversible way, the permeability and functionality of cell membranes (Toepfl et al., 2014). The two main applications of PEF to food products are the microbial inactivation (electric field in the range of 5–50 kV/cm) and the enhancement of mass transfer (electric field in the range of 0.1–5 kV/cm). In the present study, PEF pre-treatment was applied prior to brine salting to enhance mass transfer and increase salt uptake by the muscle while decreasing the duration of the process (data on salt content shown in Cropotova et al. (2021)).

Even if PEF is categorized as a non-thermal food processing technology, application of PEF-treatment can lead to a temperature increase due to the Joule effect, which must be considered with thermo-sensitive compounds such as lipids rich in polyunsaturated fatty acids (PUFAs) and proteins (Barba et al., 2015). Depending on the PEF-treatment conditions and processing parameters, the side effects of electroporation may include product discoloration due to damage to pigments, oxidation and denaturation of proteins (myoglobin), degradation and oxidation of PUFAs, bioactive peptides and vitamins, and consequently decrease of sensory characteristics and nutritional value of the end product in both plant and animal tissues (Gómez et al., 2019). Also, despite numerous studies performed on application of PEF to various food products (Chernomordik, 1992; Toepfl et al., 2014; Faridnia et al., 2015; Gómez et al., 2019), there is still a need to study the effect of this technology on oxidation reactions in muscle foods. Moreover, no studies investigating the effects of electroporation on lipid and protein oxidation reactions have to our knowledge been published.

It is very important to determine the common effects of PEF-treatment and brine salting on lipid and protein oxidation in sea bass samples. PEF-treatment prior to salting can lead to an increase in temperature during the treatment and a potential formation of free radicals acting as initiators of various oxidation reactions generating off flavors and various oxidation products causing a decrease in nutritional value through degradation of PUFAs and essential amino acids, as well as reduction of protein digestibility (Gómez et al., 2019). Electroporation can induce lipid oxidation through the formation of free radicals on unsaturated chains of fatty acids, as well as protein oxidation starting from the abstraction of a hydrogen atom from the carbon next to the amino group of the side chains. Lipid oxidation can produce rancid flavor and off-odors, thus aggravating the sensory profile of salted fish and decreasing its nutritional value, while oxidation in a side chain of amino acids can produce carbonyl groups leading to protein aggregation and loss of solubility (Rowe et al., 2004) which may also result in a decrease of water holding capacity and changes in texture of the fish muscle (Lund et al., 2011). The formation of Schiff bases can also decrease the quality of salted fish due to progressive cross-linking and polymerization reaction, including impaired functionality of myofibrillar proteins and loss of water-holding capacity (Estévez, 2011). Fish samples subjected to PEF-treatment, may exhibit greater sensitivity to lipid and protein oxidation due to higher exposure to various pro-oxidants such as heme-proteins (hemoglobin and myoglobin), transition metals and enzymes released during rupture of cell membranes (Gómez et al., 2019). The secondary lipid oxidation products may also react with primary amino groups on proteins resulting in protein carbonylation (Hematyar et al., 2019). Furthermore, a nucleophilic reaction between carbonyl groups and saturated lipid aldehydes can lead to the production of Schiff base products (Metz et al., 2004). Detection of carbonyl groups and Schiff bases are among the most common methods to detect and quantify protein oxidation in fish products (Hematyar et al., 2019).

Therefore, the aim of the present study was to investigate the combined effect of PEF-treatment and brine salting on lipid and protein oxidation in sea bass samples, as well as changes in color characteristics of the fish flesh.

## 2 Materials and methods

### 2.1 Materials

Sea bass (*Dicentrarchus labrax*) were supplied by TAGLIAPIETRA E FIGLI S.R.L. (Venice, Italy) in May 2019. The day after catch, the fish were delivered to L’ECOPESCE - ECONOMIA DEL MARE (Cesenatico, Italy) where they were gutted, filleted and deskinned. Immediately after processing, the sea bass fillets were placed on ice in Styrofoam boxes and transported to the CIRI-Agroalimentare laboratory in Cesena (Italy) where the experiment was carried out on the same day (24 h after catch).

Commercial salt "Sale alimentare di Sicilia" from ITALKALI S.RL. (NaCl ~98%) was used for preparation of brines.

### 2.2 PEF pre-treatment and brine salting

A total of 38 sea bass fillets were used for this experiment. From each fillet, 5 small pieces (8.3 ± 0.2 g each) with the dimensions of length 2.3 ± 0.2 cm, width 3.1 ± 0.4 cm and height 1.3 ± 0.5 cm were obtained (a total of 190 pieces) and randomly divided for the experimental samples. 10 pieces were used for the control sample, while 90 for each salting regime. For each salting regime, 3 sub-samples of 30 pieces were obtained and subjected to PEF pre-treatment or used as untreated ones.

**Figure 1:**
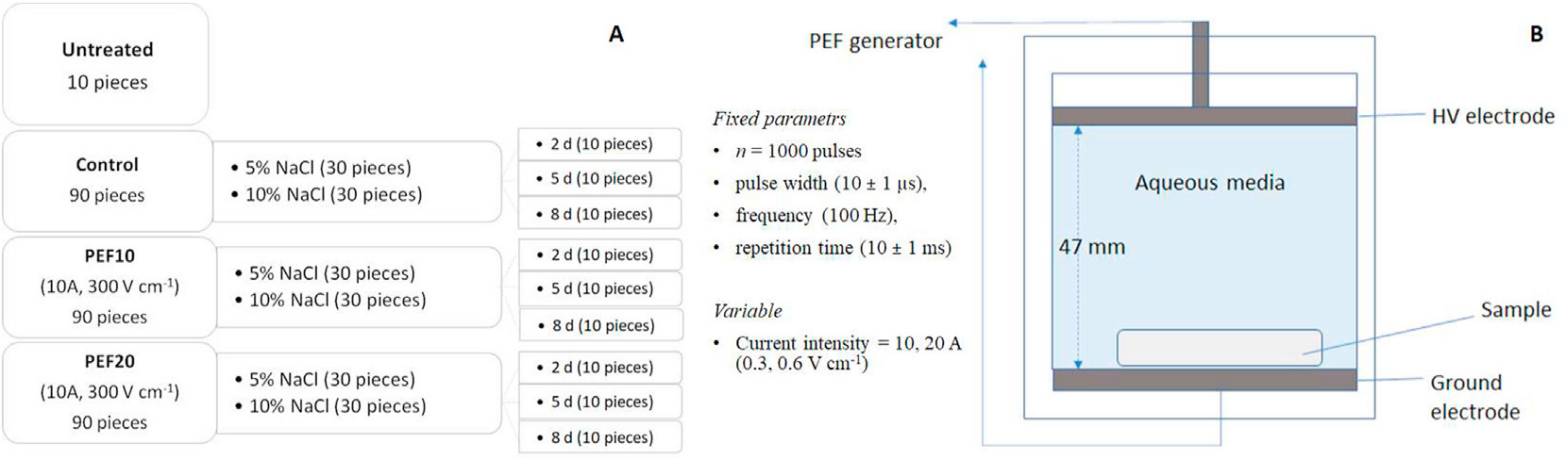
Flow chart of the process (A) and schematic representation of the treatment chamber (B).

The PEF pre-treatment, prior to salting, was performed using a lab scale PEF unit delivering a maximum output current and voltage of 60A and 8kV, respectively (Mod. S–P7500, Alintel, Italy). The generator provides monopolar rectangular-shape pulses and adjustable pulse duration (5–20 s), pulse frequency (50–500 Hz) and total treatment time (1–600 s). The treatment chamber (50 mm length x 50 mm width x 50 mm height) consisted of two parallel stainless-steel electrodes (3 mm thick) with a 47 mm fixed gap. Output voltage and current were monitored using a PC-oscilloscope (Picoscope 2204a, Pico Technology, UK). Sea bass pieces (approximately 7–8 g each) were treated at room temperature in tap water (water's initial electrical conductivity of 517 ± 20.4 μS/cm at 25 °C (EC-meter mod. Basic 30, Crison, Spain)), and delivering *n* = 1000 pulses at fixed pulse width (10 ± 1 μs), frequency (100 Hz), repetition time (10 ± 1 ms) and selecting two different current intensities, 10A and 20A, corresponding to values of electric field strengths of 300 V cm^−1^ and 600 V cm^−1^ and a total energy of 0.25 ± 0.01 and 1.01 ± 0.03 kJ/kg, respectively. The process parameters were chosen based on preliminary experimental trials, that allowed to confirm an increase in mass transfer rate halving the time necessary to reach the same salt content. The sea bass pieces were randomly distributed into the two experimental groups (PEF-treated and control samples) and salted by immersion into a brine with two different salt (NaCl) concentrations in tap water (5% and 10% w/w) at a ratio of 4:1 w/w brine/fish. Control samples were not subjected to PEF pre-treatment prior to brine salting. Samples for each sampling category were brined in one closed plastic container (5 fish pieces per one container, 500 ml). The salting process was carried out in a cold room at 0–4 °C for 2, 5 and 8 days according to the experimental plan displayed in Table 1. A flow chart of the process and a representation of the treatment chamber are shown in Figures 1A and 1B, respectively.

**Table 1:** Experimental plan.

No sample	Sample code	NaCl concentration, %w/w	Duration of salting, days	Current intensity, A	Electric field intensity, V cm^−1^
1	C-5-2	5	2	0	0
2	C-5-5	5	5	0	0
3	C-5-8	5	8	0	0
4	C-10-2	10	2	0	0
5	C-10-5	10	5	0	0
6	C-10-8	10	8	0	0
7	5-PEF-10-2	5	2	10	300
8	5-PEF-10-5	5	5	10	300
9	5-PEF-10-8	5	8	10	300
10	10-PEF-20-2	10	2	20	600
11	10-PEF-20-5	10	5	20	600
12	10-PEF-20-8	10	8	20	600
13	5-PEF-20-2	5	2	20	600
14	5-PEF-20-5	5	5	20	600
15	5-PEF-20-8	5	8	20	600
16	10-PEF-10-2	10	2	10	300
17	10-PEF-10-5	10	5	10	300
18	10-PEF-10-8	10	8	10	300

At each sampling day, sea bass samples were randomly collected and analyzed. Color measurements of the fish muscle were performed directly after each sampling day at University of Bologna (Cesena, Italy). The remaining experimental samples from each of the treatment were frozen at -80 °C and transported to Norwegian University of Science and Technology (Trondheim, Norway) for determination of lipid and protein oxidation.

Analyses were performed in 2–6 replicates for each PEF pre-treatment and salting regime.

### 2.3 Chemical and physical analyses

#### 2.3.1 Lipid extraction

Lipids were extracted from raw (control) and brine salted sea bass samples by a mixture of chloroform-methanol-water by the Bligh and Dyer (1959) method. The fish samples were minced with a kitchen blender (Bosch 600W, Gerlingen, Germany) and a sample of 10 g was taken for extraction of lipids. After centrifugation, the upper layer of lipids in chloroform extract was separated, collected and further used in primary and secondary lipid oxidation analysis.

#### 2.3.2 Primary and secondary products of lipid oxidation

The determination of primary and secondary lipid oxidation products included the quantification of peroxide value (PV), conjugated dienes (CDs) and 2-thiobarbituric acid reactive substances (TBARS), as described below.

The standard iodometric titration method (Cd 8b-90) (AOCS, 2003) was applied for PV determination. To assess the end point of titration, an automatic titrator (TitroLine 7800, Xylem Analytics, Mainz, Germany) coupled with a platinum electrode (Pt 62), was used. The titration was performed potentiometrically in duplicate and the results were expressed in meq active oxygen/kg lipids as a mean value ± SD.

CDs were determined spectrophotometrically according to the method described by Cropotova et al. (2019) as a modification of methods by Aubourg (1998) and Mozuraityte et al. (2017). Briefly, the absorbance of 1 mL chloroform extracts of lipids was measured against the solvent (chloroform) with a spectrophotometer GENESYS 12S UV-VIS (Thermo Scientific, USA) at 233 nm. The results were expressed as CD values in ml/g. The analysis was performed in two replicates for each sample, and the average with standard deviation was calculated.

TBARS were determined according to the method of Ke and Woyewoda (1979), as detailed described by Cropotova et al. (2019). For TBARS quantification, the pink-colored water phase was taken after the final step of centrifugation and its optical density was measured at 538 nm using a GENESYS 10S UV-VIS spectrophotometer (Thermo Scientifc, Pittsburgh, PA, USA). The analysis was performed in duplicate and the results were expressed in μmol TBARS/g lipids as a mean value ± SD.

#### 2.3.3 Schiff bases

Schiff bases measurements were performed according to Buege and Aust (1978) with some modifications as follows. Briefly, the fluorescence of a chloroform extract of lipids (3 mL) obtained after Bligh and Dyer (1959) extraction was measured using a luminescence spectrometer LS 50B PerkinElmer (Waltham, Ma, USA) at 360 nm excitation and 430 nm emission wavelengths. The assay was carried out in two replicates for each sample, and the results were expressed in ml/mg as a mean value ± SD.

#### 2.3.4 Protein oxidation

Protein oxidation was determined by measurement of protein carbonyls in sarcoplasmic and myofibrillar protein extracts by a DNPH based enzyme-linked immunosorbent assay (ELISA) in a 96-well polystyrene plate as described by Cropotova and Rustad (2019). The indirect ELISA kit, STA-310 OxiSelectTM, was purchased from Cell Biolabs, Inc. (San Diego, CA, USA). Sarcoplasmic (water-soluble) and myofibrillar (salt-soluble) protein extracts were prepared by a modification of the method of Licciardello et al. (1982), as previously described by Hultmann and Rustad (2002). Carbonyl groups were determined in the six parallels for each sarcoplasmic and myofibrillar protein extract, and the average value with standard deviation were calculated. The results were expressed in nmol carbonyls per mg protein.

#### 2.3.5 Color parameters

Color parameters of sea bass samples were measured instrumentally using a spectrophotocolorimeter (Colorflex, Hunterlab). Before starting the analysis, the instrument was calibrated with a standard white and black plate. The measurements were performed on two preselected locations at the surface of each sea bass piece at room temperature. The L∗, a∗ and b∗ parameters of the CIELAB scale were measured according to the lab scale established by Commission Internationale de l’Éclairage (CIE, 2001). Results were expressed as average of 10 measurements for each set of samples.

### 2.4 Statistical analysis

Statistical analysis and data processing were conducted using SigmaPlot software (Systat Software Inc., San Jose, California, USA), version 16.1.15. Statistical significance of the experimental data was verified by using Student's t-test and Analysis of Variance (ANOVA). To establish a relationship between certain parameters, Pearson correlations were calculated. Differences were considered significant at p < 0.05. The comparison analysis in ANOVA was performed by the Tukey test.

Multiple regression analysis was performed to explain the dynamics of the quality modifications in sea bass during salting and to identify the contribution of each of the process parameters to the changes occurring in the product.

## 3 Results and discussion

### 3.1 Total lipid content

**Figure 2:**
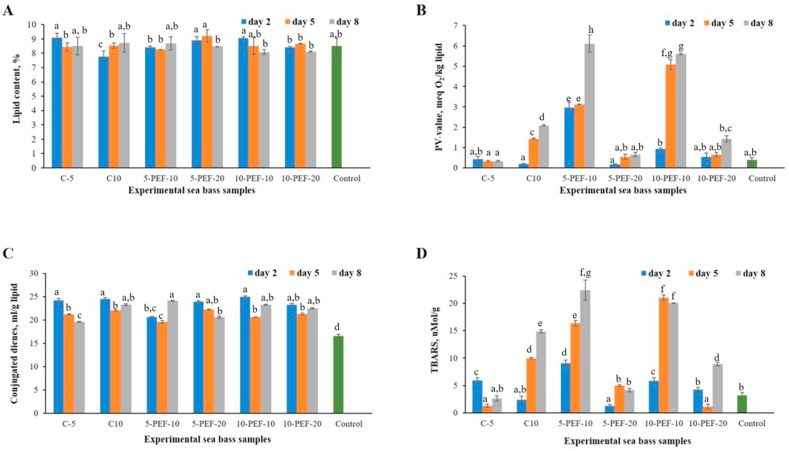
Lipid content (A) and primary and secondary lipid oxidation products: peroxide value (B), conjugated dienes (C) and TBARS data (D) of experimental sea bass samples during brine salting.

Total lipid content in brine salted sea bass samples varied from 7.78 ± 0.41 to 9.22 ± 0.43% along the experiment (Figure 2A). At the same time, no significant variation in total lipid content was found between PEF-treated and untreated fish samples over the salting period, as well as between raw (control) and salted sea bass samples.

### 3.2 Primary and secondary lipid oxidation products

Amount of primary (PV and CD) and secondary lipid oxidation products (TBARS) show that lipid oxidation increased in PEF-treated sea bass samples compared to untreated ones during brine salting (Figure 2).

The PV of sea bass samples subjected to PEF-treatment at 300 V cm^−1^ (corresponding to the current intensity of 10 A) prior to salting, displayed a significant (p < 0.05) increase compared to non-treated and PEF-treated fish samples at 600 V cm^−1^ (current intensity of 20 A) during brine salting (Figure 2B). At the same time, no significant variation in CD data was observed between PEF-treated and untreated samples (Figure 2C). However, conjugated dienes displayed a significant (p < 0.05) increase in all experimental sea bass samples along the brine salting period compared to CD-values of control samples (Figure 2C). This tendency may be because salt has been reported to enhance lipid oxidation of highly unsaturated lipids (Harris and Tall, 1994). Thus, lipids containing methylene interrupted dienes or polyenes get a shift in the position of the double bond during the oxidation due to isomerization and formation of CDs (Lalas, 2009).

However, only three sea bass samples subjected to electroporation at 10 A (300 V cm^−1^) exceeded the limit for PV of 5 meq active oxygen/kg lipid established by the Standard for fish oils CODEX STAN 329–2017 (Figure 2B) on day 5 and day 8 of brine salting. The same trend was observed in the variation of TBARS data along the salting experiment (Figure 2D). Thus, fish samples subjected to PEF-treatment at 10 A current intensity (300 V cm^−1^) had significantly (p < 0.05) higher values of TBARS compared to untreated samples and samples treated at 20 A (600 V cm^−1^).

PEF treatment prior to salting could result in destabilization of the integrity of the muscle cell membrane due to mechanical damages to the fish muscle structure, thus facilitating the exposure of various pro-oxidants (enzymes, free iron, heme-proteins, etc.) accelerating lipid oxidation. This suggestion is supported by the study of Kantono et al. (2019) who previously investigated the effects of PEF treatment on lipid oxidation in beef muscles. They believed that lipid oxidation in PEF-treated samples is associated with a breakdown of the cell membrane integrity causing a release of pro-oxidants, similar to the effect of freezing.

The phenomenon of lower lipid oxidation in samples treated with a higher field intensity (600 V cm^−1^) compared to the lower intensity (300 V cm^−1^) could be explained by an antioxidative mechanism of electroporation inactivating the pro-oxidative endogenous enzymes. A similar behavior was observed in our previous study on high pressure (HP) treatment of fish minces (Cropotova et al., 2020), according to which the lowest lipid oxidation values were obtained after the highest HP-treatment conducted at 300 MPa compared to the lowest treatment at 200 MPa. Thus, two opposite mechanisms were considered to explain the lipid changes observed: *pro-oxidative* – as a result of denaturation of heme-proteins releasing free iron and other pro-oxidants from damaged muscle cells, and *antioxidative* – leading to inactivation of pro-oxidative endogenous enzymes that could increase oxidation (Cropotova et al., 2020). Moreover, PEF inactivation of endogenous enzymes has previously been observed in different food matrixes but at significantly higher voltages (>10 kV cm^−1^) (Li et al., 2008; Liu et al., 2014; Zhao et al., 2012). However, PEF did not modify the protein profile when applied to *longissimum thoracis* beef muscles at 2–6 kV cm^−1^ (Faridnia et al., 2014) or at 1–1.25 kV cm^−1^ (Chian et al., 2019). Studies comparing the effect on microstructure of fish and meat samples showed that salmon was more sensitive to mild PEF treatment (<2 kV cm^−1^) compared to chicken. Meat composition in terms of protein and fat content and their distribution in the muscle might be the reason for these differences. However, to our knowledge, there are no studies showing the inactivation of enzymes in fish samples after PEF treatment. On the other side, the use of PEF prior to salting could have enhanced the distribution of salt in the tissue thus promoting protein aggregation. Hence, this behavior should be further investigated.

### 3.3 Lipid-protein and protein oxidation products

**Figure 3:**
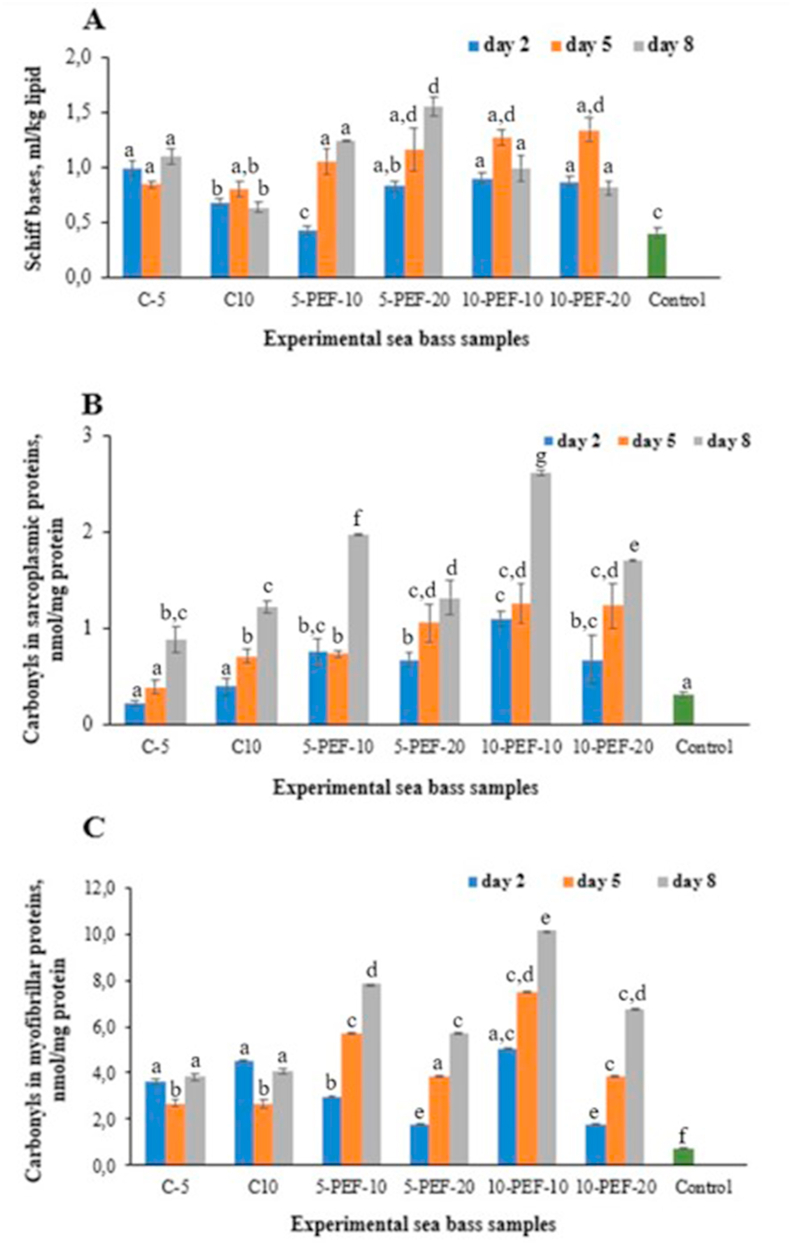
Products of lipid-protein and protein oxidation reactions in experimental sea bass samples: Schiff bases (A) and protein carbonyls in sarcoplasmic (B) and myofibrillar (C) proteins.

The end products of lipid oxidation can react with proteins, thus affecting the color and flavor of the fish and reducing nutritional value of the proteins. Schiff bases (SB) formation is one of these reactions resulting from a cross-linking between the aldehyde moiety from protein carbonyls and alkaline amino acids in proteins (Estévez, 2011). Assessing the effect of the PEF treatment on formation of SB in salted fish can be a valuable tool to optimize the process and reduce cross-linking reactions which can cause protein polymerization and impaired functionality, including loss of water-holding capacity. In the present study, there was a significant (p < 0.05) increase in SB content in all experimental sea bass samples compared to raw fish (Figure 3A).

**Figure 4:**
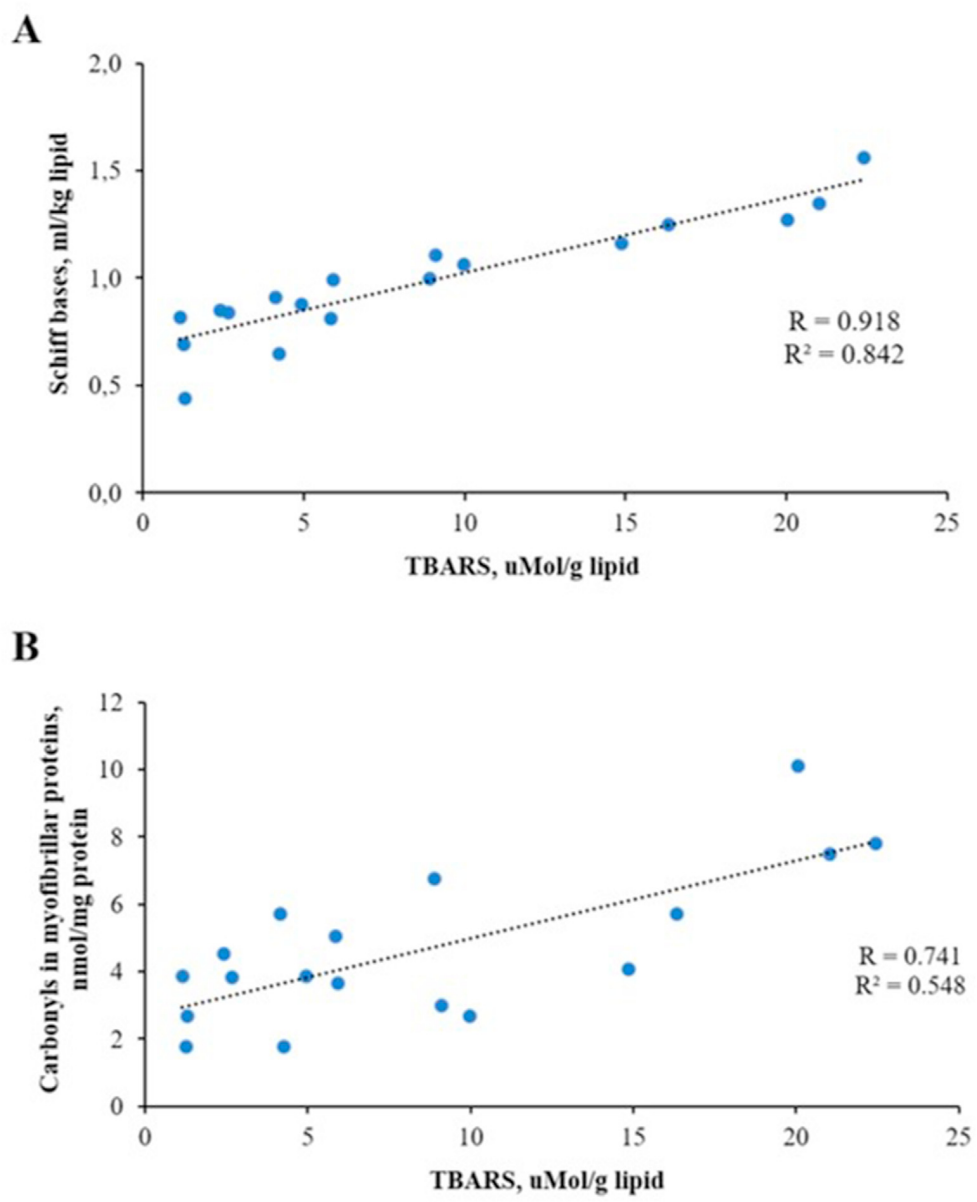
Correlations between lipid and protein oxidation products: Schiff bases and TBARS (A) and carbonyls in myofibrillar proteins and TBARS (B).

At the same time, all PEF-treated fish samples showed a significant increase in Schiff bases values on day 5 and day 8 of brine salting compared to non-treated salted sea bass (Figure 3A). Contrarily to results related to fat oxidation, the fish samples salted in 5% brine had significantly higher values of Schiff bases after PEF treatment conducted at higher current intensity (20 A) compared to samples subjected to electroporation at lower current intensity (10 A). The end products of lipid and protein oxidation interact with each other, including a nucleophilic reaction between carbonyl groups and saturated lipid aldehydes producing Schiff bases (Metz et al., 2004). In support to this suggestion, a significant correlation (p < 0.05, R = 0.918) between TBARS and SB of experimental sea bass samples during brine salting, was revealed (Figure 4A).

To the best of our knowledge no information about the effects of electroporation on protein oxidation in fish is available in the literature. Moreover, the existing information on the influence of PEF-treatment on protein oxidation in various protein-rich food products is scarce and mostly includes studies on eggs and egg products carried out at much higher electric field strength (Wu et al., 2014). Fish is a rich and important source of proteins with bioactive properties, which may be prone to denaturation, aggregation and oxidation when exposed to electroporation, resulting in decline of product quality and nutritional value (Gómez et al., 2019). Therefore, assessment of protein oxidation in the product can be a valuable tool for further optimization of the process parameters (applied electric field intensity, salt concentration, etc.).

Our investigations revealed a significant (p < 0.05) increase in total carbonyls in sarcoplasmic and myofibrillar proteins of PEF-treated sea bass samples compared to control and untreated samples on day 5 and 8 of brine salting (Figure 3B-C). This phenomenon can be explained by PEF-induced degradation of cell membranes due to mechanical damages to the fish muscle structure under the applied current of 10 and 20A (300 and 600 V cm^−1^), leading to the release of various pro-oxidants (enzymes, free iron, heme-proteins, etc.) accelerating protein oxidation reactions (Gómez et al., 2019). Secondary lipid oxidation products can also react with primary amino groups on proteins resulting in protein carbonylation (Hematyar et al., 2019). This is supported by the significant correlation (p < 0.05, R = 0.741) found between carbonyls in myofibrillar proteins and TBARS, (Figure 4B).

### 3.4 Color parameters (yellowness)

**Figure 5:**
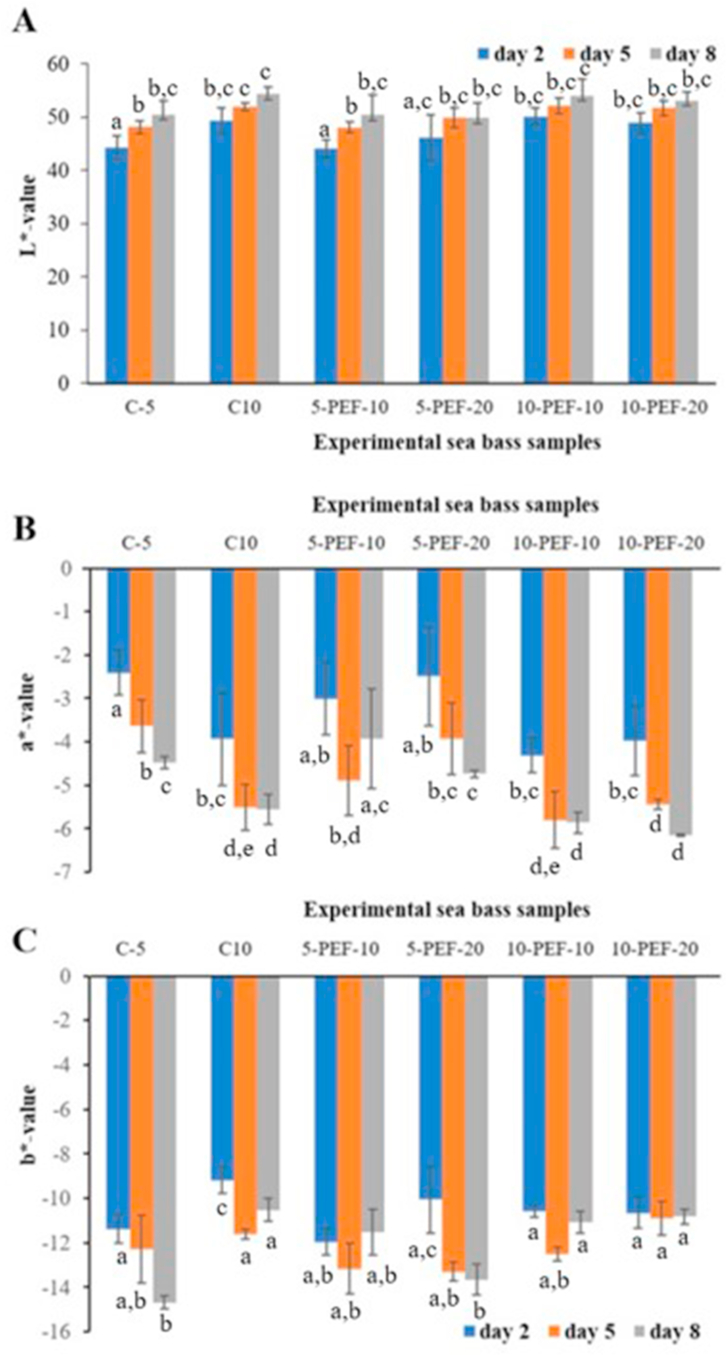
Color characteristics of sea bass samples during brine salting: lightness (L∗-value) (A), redness (a∗-value) (B), and yellowness (b∗-value) (C).

According to Figure 5A, lightness of experimental sea bass samples varied from 44.2 to 54.2, while increasing gradually over the duration of salting period. Multiple regression analysis revealed that the main factor influencing the changes of L∗-value was duration of salting period (p < 0.05, R = 0.974, R^2^ = 0.951). However, no significant difference between PEF-treated and untreated sea bass samples was found during brine salting. At the same time, the increase in lightness was accompanied by a simultaneous decrease in redness in sea bass samples during brine salting (Figure 5B). Moreover, there was a significant (p < 0.05) decrease in a∗-value in PEF-treated samples compared to untreated ones during brine salting, indicating that fish loses its natural flesh pigmentation when subjected to electroporation – a trend similar to cooking discoloration. This phenomenon can be explained by denaturation of some pigments and heme-proteins inside the fish muscle (Gómez et al., 2019). A similar effect was observed in our previous study on high pressure treatment of Atlantic mackerel and haddock minces (Cropotova et al., 2020) suggesting that changes in color characteristics are due to conformational changes in heme-proteins. Based on previous investigations, we hypothesize that the gradual decrease in redness of PEF-treated sea bass samples compared to untreated ones during brine salting is mainly due to PEF-induced denaturation of heme-proteins inside the fish muscle.

Yellowness of experimental sea bass samples displayed a high variation throughout the storage period (Figure 5C). The changes in b∗-value of brine salted sea bass are most likely due to accumulation of yellowish-colored compounds generated by decomposition and polymerization of primary products of lipid oxidation, indicated by the positive correlation values (R) of 0.789 (p < 0.05) between b∗-values and conjugated dienes.

## 4 Conclusion

The present study investigated the effect of PEF treatment prior to brine salting on lipid and protein oxidation and changes in color characteristics of sea bass. Electroporation negatively influenced the oxidative lipid stability in sea bass samples with regard to primary and secondary lipid oxidation products.

Damage of cell membranes occurring during electroporation resulted in higher oxidation level with respect to primary and secondary oxidation products and protein carbonyls of PEF-treated sea bass samples compared to untreated fish samples during brine salting. However, sea bass samples treated at higher voltage of 600 V cm^−1^ showed significantly lower values of peroxide value and TBARS compared to fish samples treated at 300 V cm^−1^. At the same time, none of the untreated and PEF-treated samples at the voltage of 600 V cm^−1^ exceeded the acceptable level of 5 meq active oxygen/kg lipids suggesting satisfactory quality of sea bass during brine salting with regard to oxidative lipid stability.

PEF-treated sea bass samples were also characterized as lighter and less reddish compared to untreated fish samples. The yellowness of sea bass samples correlated significantly with conjugated dienes, suggesting the contribution of electrochemically polymerized lipids on further generation of secondary lipid oxidation products leading to yellowing of the fish flesh.

The present study showed that it is important to consider and select PEF-treatment process parameters prior to brine salting in order to obtain the advantages due to the PEF application on salting kinetics but to avoid extensive lipid and protein oxidation in the fish during the salting process.

## Declarations

### Author contribution statement

Janna Cropotova: Conceived and designed the experiments; Performed the experiments; Analyzed and interpreted the data; Contributed reagents, materials, analysis tools or data; Wrote the paper.

Silvia Tappi: Performed the experiments; Analyzed and interpreted the data; Contributed reagents, materials, analysis tools or data; Wrote the paper.

Jessica Genovese: Performed the experiments; Analyzed and interpreted the data; Wrote the paper.

Pietro Rocculi: Analyzed and interpreted the data; Contributed reagents, materials, analysis tools or data; Wrote the paper.

Marco Dalla Rosa: Contributed reagents, materials, analysis tools or data; Wrote the paper.

Turid Rustad: Conceived and designed the experiments; Contributed reagents, materials, analysis tools or data; Wrote the paper.

### Funding statement

This work was supported by the International Research Mobility Support offered as part of NTNU Postdoc Action Pilot Programme and EU project FuturEUAqua H2020-BG-2018-2020 (Blue Growth).

### Data availability statement

Data will be made available on request.

### Declaration of interests statement

The authors declare no conflict of interest.

### Additional information

No additional information is available for this paper.
